# Supplementary material for: The interactome of intact mitochondria by cross-linking mass spectrometry provides evidence for coexisting respiratory supercomplexes
Source: Mol Cell Proteomics. 2017 Dec 8;17(2):216–32. doi: 10.1074/mcp.RA117.000470 (PMC5795388; doi:10.1074/mcp.RA117.000470)
Supplement: Supplemental Data [file supp_RA117.000470_133922_0_supp_23978_fzffwf.zip › spectra_annotation/mito_spectra_annotation/4-1-1-1-23-1.pdf]

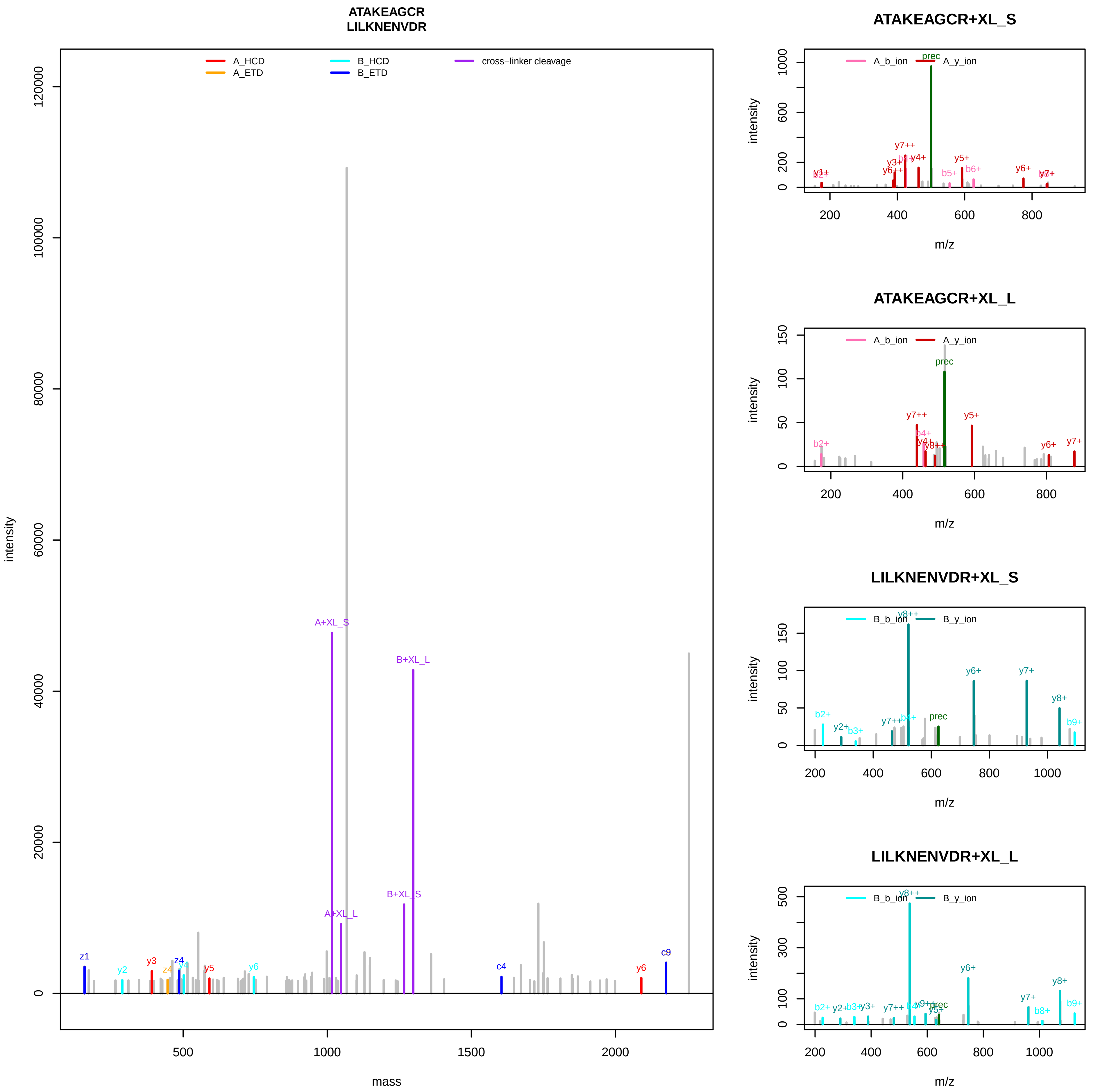

ATAKEAGCR
LILKNENVDR
A_HCD
A_ETD
B_HCD
B_ETD
cross−linker cleavage
intensity
mass
A+XL_S
B+XL_L
B+XL_S
A+XL_L
z1
y2
y3
z4
z4
y4
y5
y6
c4
y6
c9
ATAKEAGCR+XL_S
A_b_ion
A_y_ion
prec
y7++
y3+
y6++
y4+
y5+
b5+
b6+
y6+
y7+
y1+
m/z
ATAKEAGCR+XL_L
b2+
b4+
y4+
y8++
y6+
LILKNENVDR+XL_S
B_b_ion
B_y_ion
y8++
b2+
y2+
b3+
y7++
b4+
y6+
y7+
y8+
b9+
LILKNENVDR+XL_L
b8+
y9++
y5+
